# Wake up! Voluntary Hospital Managers

**Published:** 1911-08-12

**Authors:** 


### The Hospital

A Journal of

The Medical Sciences and Hospital Administration.

New Series. No. 237, Vol. IX. [No. 1309, Vol. L.] SATURDAY, AUGUST 12, 1911

### Wake up! Voluntary Hospital Managers.

We regret to have to point out that the cause of the voluntary hospitals found no champion in the House of Commons when Clause 15, the important clause so far as voluntary hospitals are concerned, was under discussion. Dr. Hillier had important amendments on the paper which would have protected the voluntary hospitals and secured their interests, but none of them was adopted and, so far as the public proceedings in Parliament are reported, it would appear that none of them was even moved. Thus the House of Commons has shown a marked disregard for, and almost a lamentable absence of interest in, the welfare of the voluntary hospitals, facts which we commend to hospital managers generally, for the responsibility of accepting or altering this regrettable state of affairs now rests mainly with them. We will come to the cause which underlies this neglect directly, but the first point to drive home is that the voluntary hospitals at present are not sufficiently organised to make their interests and work weigh with the members of the present House of Commons.

When men become members of the House of Commons it is supposed that their first object is to further the interests of all their constituents whose representatives they in fact are. If the interests and welfare of the sick and suffering poor all over the country are not regarded as among the first of such duties by members of the present House of Commons, they must differ very much for the worse from the type of public representative who used to be sent up, by English constituencies at any rate. Mr. Lloyd George has been worried and harassed, no doubt, but we hoped from what he told us himself and from his reception of the deputations from the hospitals, that he would take care that the voluntary hospitals did not suffer under his Bill. Be this as it may, he has not yet done anything whatever to lighten the burden of depletion of income which his Insurance Bill will cast upon the voluntary hospitals. Disappointment is the inheritance of zealous workers for others all the world over, and voluntary hospital managers *vis-à-vis* with politicians must regard, with philosophy, no doubt, the slight cast upon their work by the House of Commons. We, however, make bold to think and to say that the shabby treatment the voluntary hospitals have so far received in this matter should stir up every hospital worker throughout the United Kingdom, and that all should combine and organise a concerted effort to bring Parliament to its senses. We must move Parliament to secure adequate protection for the sick poor against the serious disasters and avoidable sufferings and miseries which the Insurance Bill as it stands must bring to many of the most deserving and suffering amongst them.

The steps to be taken are simple, but plain. Every city and town containing a voluntary hospital or hospitals should form a committee representative of all the hospitals, who should appoint an active chairman and secretary to set to work steadily to bring to the knowledge of the inhabitants the ruinous effect which this Bill may have upon the present voluntary hospital provision. They should further organise meetings of protest, and between now and the assembling of Parliament in the autumn, consolidate the opinion of all the most representative men amongst them, and get them, when the Insurance Bill is next considered, to make it plain to every representative in Parliament, whatever his politics may be, that he must bestir himself and see that the voluntary hospitals are safeguarded under the Bill. The necessary changes to effect this object can be introduced at the report stage, but to ensure this the managers of voluntary hospitals must wake up and organise public opinion everywhere, for private representation, backed by facts, has so far failed to move Mr. Lloyd George or those responsible for the wording of the Bill.

Most members of the present House of Commons no doubt would declare if asked that they had the keenest personal interest in the voluntary hospitals, and that they would leave nothing undone to help them in their beneficent work. Yet the procedure in the House of Commons nowadays, where private members have practically neither voice nor influence, necessarily defeats all practical legislation which is not backed by the prevailing majority. The real cause why no amendments were moved to Clause 15 probably was that this clause came on between 2 and 3 A.M., before a weary and jaded House, which was temporarily deprived of the presence of some of its ablest members, owing to the fatigue of having sat there continuously for many hours and the necessity of obtaining refreshment. This is the explanation which has been given to us by one of the ablest and most experienced members of the present House of Commons. It is one necessary result of undue rush and hurry. Our reply must be that those in charge of the Bill, in view of the deputations which have waited upon them, and of the representations which have been made to the Chancellor of the Exchequer and his reception of such representations, should have taken steps to see that the cause of the hospitals was not sacrificed by hurrying through this clause in the circumstances just mentioned. The more closely this neglect of the voluntary hospitals is looked into, the more certainly will it arouse feelings of indignation, surprise, and regret amongst all classes of the community. There is yet time to set the matter right on report, but if the voluntary hospitals are to be adequately protected the managers of these institutions must be up and doing by the middle of September. Then they must continuously work each in his own locality to influence the local members of Parliament, and all collectively through the British Hospitals Association, by bringing pressure to bear upon the Government, to secure that the very moderate alterations which we have advocated shall be inserted. It will never do to accept Mr. Lloyd George's optimism, which led him and Mr. Sydney Holland to advise that the voluntary hospitals should wait and see what damage the passing of this Bill will inflict upon their incomes. When this loss arose then would be the time to wake up and ask the Government to make up any such deficiency. The best interests of the sick and suffering poor, or the equally grave issues which depend upon the efficient maintenance of the voluntary hospitals, cannot be allowed to be left in doubt. To lock the door after the steed has been stolen means, in the case of the voluntary hospitals, permanent disablement. Indeed, if the managers do not now exert themselves by taking vigorous individual and united action, upon them must rest the chief responsibility for any disaster which may arise from the passing of the Insurance Bill as it stands at present. Some of the best men in the hospital world have worked hard at this question already, and we look to them and to every hospital manager all over the United Kingdom to wake up and put his shoulder to the wheel, that the voluntary hospitals may be neither crippled nor injured, but, on the contrary, that they may be protected and maintained with even greater efficiency in the future than in the past.

